# Duration of insomnia and success expectancy predict treatment outcome of iCBT for insomnia

**DOI:** 10.3389/frsle.2024.1415077

**Published:** 2024-11-06

**Authors:** Polina Pchelina, Mikhail Poluektov

**Affiliations:** ^1^Cereneo, Center for Neurology and Rehabilitation, Vitznau, Switzerland; ^2^Department of Neurology and Neurosurgery, I.M. Sechenov First Moscow State Medical University, Moscow, Russia

**Keywords:** chronic insomnia, internet-based cognitive behavioral therapy for insomnia, Insomnia Severity Index, responders, predictors, multiple imputation

## Abstract

**Introduction:**

Identifying prognostic factors of treatment outcome may assist in customizing an intervention to a patient's needs. Hence, we conducted a secondary analysis of data from a randomized controlled trial to investigate the effectiveness of an internet-based cognitive behavioral therapy for insomnia (iCBT-I) to find patient characteristics that may predict the change of insomnia severity after treatment.

**Materials and methods:**

In this exploratory analysis involving 94 chronic insomnia patients, we examined the predictive value of several self-reported measures, medical history, and sociodemographic variables to psychological distress with separate linear regression models. The main outcome was the Insomnia Severity Index score improvement from pre- to post-treatment

**Results:**

The study found that duration of insomnia, *b* (*SE*) = −0.02 (0.01), *p* = 0.01, and attitudes about the expected treatment success, *b* (*SE*) = 0.80 (0.27), *p* = 0.004, were predictors of a better outcome. Moreover, a better outcome was associated with a lower level of the following traits: attention seeking, *b* (*SE*) = −1.06 (0.51), *p* = 0.04; grandiosity, *b* (*SE*) = −1.50 (0.57), *p* = 0.01; distractibility, *b* (*SE*) = −1.57 (0.75), *p* = 0.04; and rigid perfectionism, *b* (*SE*) = −1.32 (0.65), *p* = 0.05.

**Conclusion:**

Our results suggest that iCBT-I might be particularly beneficial for patients with higher expectations from the therapy and those who have a shorter duration of insomnia. Some pronounced personality traits, such as attention seeking, grandiosity, distractibility, and rigid perfectionism, may predict worse outcomes. However, because this was a *post-hoc* analysis, our results must be considered exploratory and verified in further studies.

**Clinical trial registration:**

https://clinicaltrials.gov/study/NCT04300218?cond=NCT04300218&rank=1, Identifier NCT04300218.

## 1 Introduction

Chronic insomnia (CI) is a common, burdensome sleep disorder that affects 10% of adults in the global population (Baglioni et al., 2020; Ohayon and Reynolds, 2009). CI significantly impairs an individual's quality of life, functioning, and overall health. It is associated with a range of adverse consequences, including a higher risk of physical and mental health impairment, reduced work productivity, and an elevated risk of accidents (Baglioni et al., 2016; Overton et al., 2023; Chellappa and Aeschbach, 2022; Silva et al., 2022). Cognitive-behavioral therapy for insomnia (CBT-I) is a gold-standard intervention, having demonstrated efficacy in both clinical and real-world settings (Dieter et al., 2017; Wilson et al., 2019; Edinger et al., 2021). This therapeutic approach focuses on modifying dysfunctional cognitions and maladaptive behaviors that perpetuate sleep disturbances. Traditionally, CBT-I has been delivered during individual face-to-face sessions, although a lack of knowledge about this approach among patients and clinicians, a shortage of trained CBT-I clinicians, and difficulties in accommodating optimal times and suitable locations for therapy sessions make this method unavailable for a broad patient population (Riemann et al., 2022). Surveys show that primary care doctors tend to prescribe pharmacological treatment because they lack the time to provide regular CBT-I support in routine practice, have poor knowledge about CBT-I, and sometimes have no trained CBT-I specialists in the area (Linder et al., 2021; Everitt et al., 2014). Hence, the more accessible internet-based self-management interventions have become a promising alternative to the traditional CBT-I approach.

Several meta-analyses and reviews have shown that 60–70% of CI patients can benefit from internet-based CBT-I (iCBT-I) programs with or without therapist guidance (Seyffert et al., 2016; Zachariae et al., 2016). However, online approaches, as is also the case of face-to-face CBT-I, do not produce significant improvement in 30–40% of chronic insomnia patients (Ritterband et al., 2017; Morin et al., 2002). This causes a need for a more nuanced understanding of the factors that predict treatment outcomes in clinical practice. Depending on these factors, patients may be referred to a less tailored but more accessible treatment (manualized CBT-I delivered by a trained general practitioner or an iCBT-I application and program) or to a more individually tailored therapy delivered by a sleep medicine expert according to a stepped-care approach to insomnia medical care (Baglioni et al., 2020; Espie, 2009).

Demographic variables, such as sex, age, education, outcome expectancy, comorbid depression, and anxiety, have predicted treatment effects in a series of studies (Yeung et al., 2015; Batterham et al., 2017; Blom et al., 2015; Pchelina et al., 2023). However, subsequent studies in a similar context failed to replicate these findings. The most stable predictive effects are observed for emotions, maladaptive cognitions, and sleep-related behavior: sleep-threat monitoring, dysfunctional beliefs, safety behaviors, sleep-related worry, and pre-sleep arousal (Batterham et al., 2017; Gosling et al., 2018). Certain personality traits may predispose, accentuate, and perpetuate insomnia or interact with patient–therapist relationships and therapeutical techniques, in many ways alleviating or affecting them. Previous studies have shown that insomnia symptoms and treatment outcomes are reliably associated with negatively oriented and maladaptive personality traits, such as neuroticism, perfectionism, reward dependence, and obsessive-compulsive traits (Akram et al., 2023; Lee et al., 2012; Petrov et al., 2020). By comparison, perfectionism can be adaptive to a certain degree because it manifests in the discipline, greater organization, and personal standards needed to stand the CBT-I recommendations (Lee et al., 2012; Johann et al., 2023). Reward dependence is another trait that has a proven positive effect on treatment engagement and adherence and reduces the risk of dropout (An et al., 2012). As knowledge about the predictors of iCBT-I treatment efficacy is scarce and sometimes even contradictive, in this exploratory study, we aim to find CI patients' characteristics that may predict the change of insomnia severity after iCBT-I.

## 2 Materials and methods

**Figure 1:**
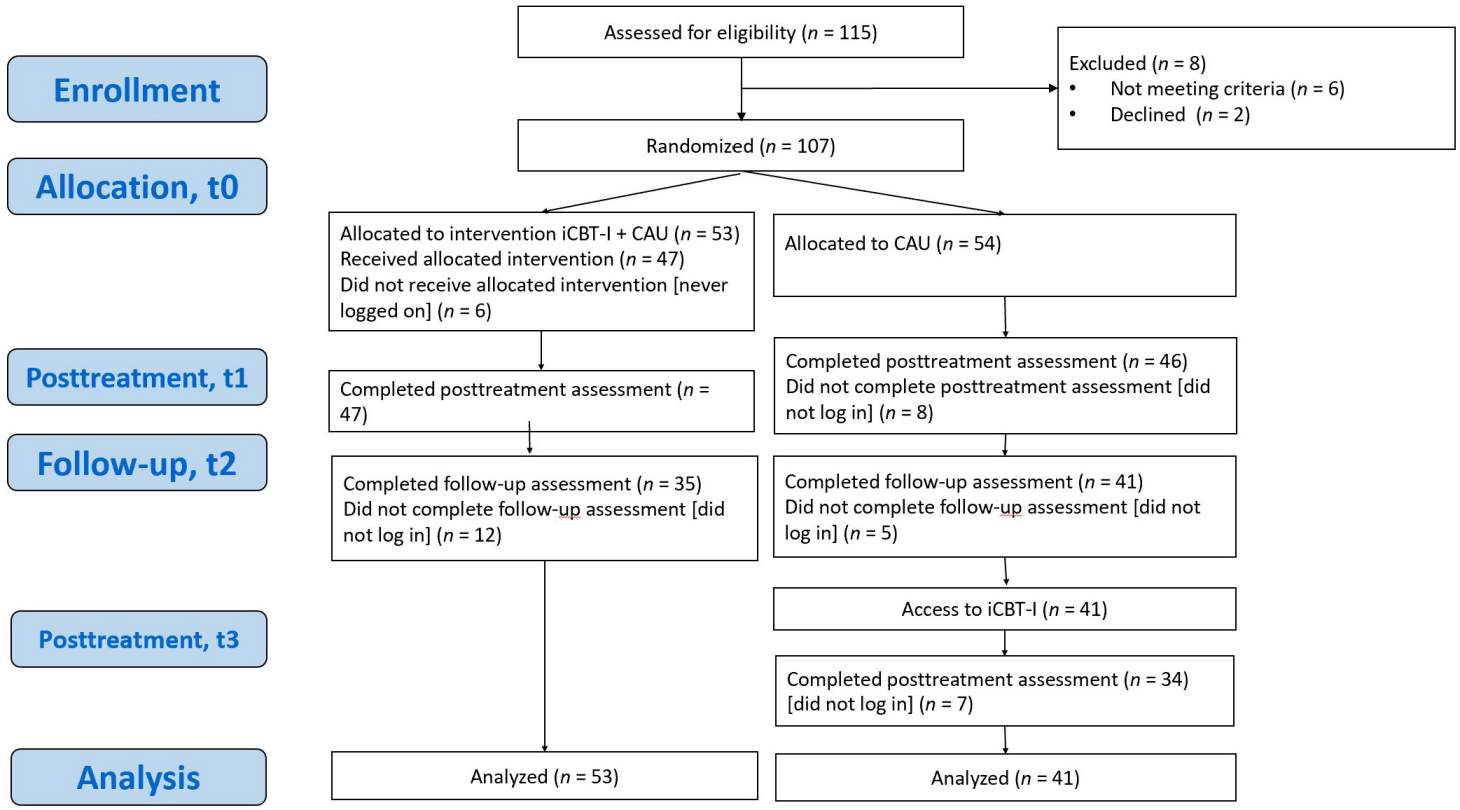
Participant flowchart. iCBT-I, internet-based cognitive behavioral therapy for insomnia; CAU, care as usual.

Data for this analysis were collected in a parallel-group randomized controlled trial comparing participants in an iCBT-I + care-as-usual (CAU) group with participants in a CAU-alone group in a clinical setting. CAU means that participants could receive a specific treatment for insomnia based on a doctor's decision. Participants (*N* = 107, age range: 18–80 years) with CI according to the *International Classification of Sleep Disorders–Third Edition* (American Academy of Sleep Medicine, 2014) were recruited in three outpatient sleep medicine centers based in Russia—the sleep medicine department at University Clinic 3, Sechenov First Moscow State Medical University, Moscow (*n* = 109); the Stavropol regional clinical sleep center (*n* = 3); and Kuzbass Clinical Hospital for Veterans (*n* = 3)—from March to December 2022. Patients were not included if they had severe depressive or anxiety symptoms as assessed using the Beck Anxiety Inventory (BAI) and the Beck Depression Inventory–II (BDI-II), had psychiatric conditions distinct from depression and anxiety, and had other sleep, neurological, or somatic disorders that affect night sleep. After signing an informed consent form, participants completed online baseline questionnaires and were automatically assigned to either the iCBT-I + CAU or the CAU group using single-block randomization with a 1:1 allocation ratio. The detailed protocol of the study recruitment, intervention description, and results of the primary analysis have been previously reported (Pchelina et al., 2020, 2024). The trial was registered at clinicaltrials.gov (NCT04300218) and was approved by the local ethics committee of I.M. Sechenov Moscow Medical University (No. 03-20/19.02.2020). The investigated 8-week guided iCBT-I program consisted of participants completing a sleep diary and 8 modules that included video lectures, tasks based on psychoeducational, behavioral (bedtime restriction, stimulus control, and relaxation), and cognitive (cognitive restructuring) techniques. Subsequent online assessments were conducted at week 8 (t1, posttreatment) and week 20 (t2, follow-up) for both groups. After completing the follow-up assessment, participants in the CAU group were granted access to the iCBT-I program. Following program completion, they additionally filled in online questionnaires at week 28 (t3, posttreatment). The participant flowchart is presented in Figure 1.

### 2.1 Outcome measures

The Insomnia Severity Index (ISI) is a seven-item insomnia assessment tool that examines both night- and daytime aspects of insomnia disorder and is sensitive to treatment response. The 5-point Likert scale is used to rate each item (e.g., 0 = *no problem*; 4 = *very severe problem*), yielding a total score ranging from 0 to 28, with higher scores indicating more severe insomnia. The Russian version of the ISI has shown acceptable psychometric properties (Bastien et al., 2001; Morin et al., 2011; Rasskazova, 2008). The primary outcome of the present analysis, ISI score improvement, was calculated as the arithmetical difference between ISI score pretreatment and ISI score posttreatment; that is, higher values of ISI score improvement were indicative of more favorable treatment outcomes. Per the protocol timing of the iCBT-I treatment, to get the ISI score improvement for the iCBT-I + CAU group, we subtracted the ISI score at t1 from the ISI score at t0 to get the ISI improvement; for the CAU group, we subtracted the ISI score at t3 from the ISI score at t2 (see Figure 1). The ISI score improvement was further categorized based on a cutoff value of ISI score improvement ≥8, defined as a sensitive and specific criterion of treatment response in Morin et al.'s (2011) work, and a resulting binary variable (response and non-response) was used as a secondary outcome measure to complement the findings (Morin et al., 2011). The decision to use two outcomes (continuous and categorical) was made to combine the higher reliability and the statistical power of continuous outcome with the categorical outcome, which simplifies interpreting the results, which is more useful in clinical practice.

We included baseline demographic and medical history characteristics as potential predictors of the outcome. Age and duration of insomnia were treated as continuous variables. Sex, concurrent pharmacotherapy, and therapy with benzodiazepines were presented as categorical variables with two levels. Social status, level of education, employment status, and the presence of comorbid diseases were coded as categorical variables with four levels. Other potential predictors measured at pretreatment were the BAI and the BDI-II (Beck et al., 1988, 1996; Ivanec et al., 2016), the quality-of-life 12-Item Short-Form Survey (SF-12) version 1.0 (Ware and Sherbourne, 2012; Novik and Ionova, 2002), the Fatigue Severity Scale (Krupp et al., 1989), the Epworth Sleepiness Scale (Johns, 1991), the Sleep Hygiene Index (SHI) (Mastin et al., 2006), the Sleep Locus of Control Questionnaire, (Rasskazova, 2008; Vincent et al., 2004), and the Dysfunctional Beliefs and Attitudes About Sleep Scale (DBAS) (Rasskazova, 2008; Morin et al., 2007). Weekly average subjective sleep characteristics—sleep efficiency (SE), total sleep time (TST), sleep onset latency (SOL), and wake time after sleep onset (WASO)—were derived from the participant's sleep diary. To analyze the predictive value of personality traits, we included the Personality Inventory for DSM-5 Faceted Brief Form (PID-5-FBF) 100-item self-report inventory designed to assess the pathological personality trait facets and the five domains based on the dimensional trait model (*Diagnostic and Statistical Manual of Mental Health Disorders, Fifth Edition*, Section III) in the baseline assessment (Maples et al., 2015; Miller et al., 2022). To assess the predictive value of attitudes about the expected treatment success, one question, using a 1–9 scale, was adapted from the Credibility/Expectancy Questionnaire: “At this point, how successful do you think this treatment will be in reducing your insomnia symptoms?” This question was chosen because it featured a high correlation for both factors, credibility and expectancy, and was most logically formulated for the intended purpose (Devilly and Borkovec, 2000).

All assessments were administered online on the Qualtrics Survey platform and consisted of self-report questionnaires. We used the Russian-language validated versions of the self-report questionnaires if they were available. Forward and backward translation for the SHI and PID-5-FBF questionnaires was conducted because no validated Russian version was available. For the present secondary analysis, data from both groups were combined using data from different time points. Sociodemographic and medical history variables, as well as outcome expectancy and personality traits [evaluated by Personality Inventory for Diagnostic and statistical manual of Mental Disorders, 5th edition (PID-DSM-V)] were collected for both groups at baseline (t0). The other predictors of posttreatment outcome measured using several self-report scales (the BDI-II, the BAI, the DBAS, etc.) were assessed before the respective treatment phase, that is, for the iCBT-I + CAU group at baseline, t0, and the CAU group at follow-up, t2 (see Figure 1). Adherence was assessed using elements of iCBT-I: the number of completed modules, the number of completed sleep diaries, and the number of emails sent to the iCBT-I specialist during the program. These variables were analyzed as potential mediators of the outcome.

### 2.2 Statistical analysis

Statistical analysis was performed with R (R Core Team, 2016). Independent samples *t*-tests for continuous normally distributed variables, the Mann–Whitney test for continuous non-normally distributed variables, and χ^2^ tests for categorical data were performed to examine differences between the iCBT-I + CAU and the CAU group at baseline and pretreatment. Because the pretreatment ISI score was significantly different between the two groups (*p* = 0.04) and explained a large fraction of the variance of the final score, it was included in all models. For each potential predictor, a separate linear regression analysis was performed as follows: The potential predictor was entered as a predictor, the posttreatment score of the outcome (ISI score improvement or response) was entered as a dependent variable, and the pretreatment ISI score was defined as a statistically and clinically significant covariate. To account for possible group effects, we additionally tested whether the group (iCBT-I + CAU vs. CAU) was a significant predictor by adjusting the models for group effects. The models were tested using the *F*-statistic and Akaike/Bayesian information criteria to select the best one. Models adjusted for group effect and ISI^*^group interaction effect did not outperform the model including only the baseline ISI score. The final model looked as follows: ISI_improvement ~ ISI_pre + Var_predictor, where “ISI_improvement” was the main outcome, “ISI_pre” was the pretreatment ISI score, and “Var_predictor” was used for every assessed predictor.

**Figure 2:**
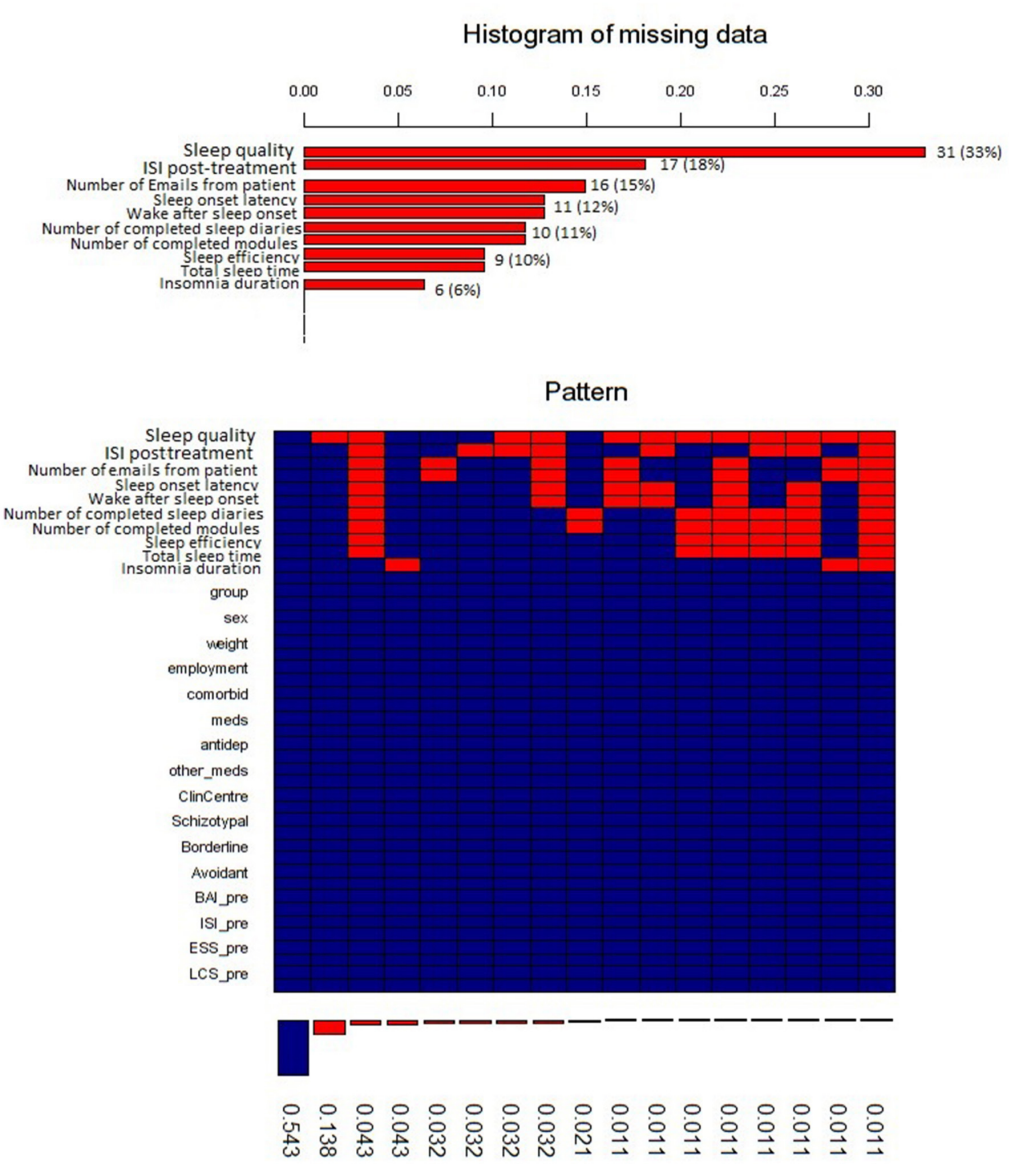
Histogram of the missing data. ISI, Insomnia Severity Index; meds, therapy with any medications; antidep, therapy with antidepressants; other_meds, therapy with other medications; ClinCentre, clinical center; Schizotypal, Schyzotypal type of personality disorder in accordance to AMPD; Borderline, type of personality disorder in accordance to AMPD; Avoidant, avoidant type of personality disorder in accordance to AMPD; BAI, Beck anxiety inventory pretreatment; ISI_pre, Insomnia severity index pretreatment; ESS_pre, Epworth sleepiness scale pretreatment; LCS_pre, Locus control of sleep scale pretreatment.

The percentage of missing values across the investigated predictor variables varied between 0 and 33% (Figure 2). Among participants, 17 (18%) did not complete the posttreatment assessment, including the ISI. Because of the small sample size, we decided to use multiple imputation instead of excluding incomplete cases. Multiple imputation is considered a state-of-the-art technique for handling missing values because it improves the accuracy and statistical power relative to other missing data techniques (Wulff and Ejlskov, 2017). We used R package mice with the imputation method “cart” (classification and regression trees), a number of multiple imputations equal to 5, and a number of iterations equal to 50 to create and analyze 5 multiply imputed data sets (Schafer, 1997; van Buuren and Groothuis-oudshoorn, 2011). Because the rate of missing data was 15% for ISI improvement posttreatment and 28% for ISI score improvement at follow-up, multiple imputations were used for all the statistical models. Robustness and accuracy of the imputed data set were checked with the imputed data's cross-validation using linear regression and “caret” package in R. This analysis showed a good model fit, with Root Mean Squared Error (RMSE) < 0.001, R-squared = 1, Mean Absolute Error (MAE) < 0.001 (Kuhn, 2008). For robustness, a complete case analysis was performed, and the results of the models built from the observed and imputed data sets were compared. We report data from both the imputed and incomplete data sets, but the imputed data set was prioritized because multiple imputations allow for more accurate and precise estimates of the parameters of interest (Hayati Rezvan et al., 2015; Sterne et al., 2009).

## 3 Results

For the primary study, we enrolled 107 participants, but 13 participants in the CAU group dropped out by the end of follow-up/start of treatment. Therefore, the total sample in this exploratory study consisted of 94 participants. The median age was 40 years (range = 18–81 years); the majority were female (*n* = 54, 57.4%) and had a university degree (*n* = 79, 84%). Overall, 79 participants (84.0%) were taking medication for insomnia at baseline. Baseline or pretreatment scores of the predictor variables and outcome measures are displayed in Table 1. Pretreatment, there was a significant group difference in terms of age, ISI scores, SHI scores, and SF scores. Personality traits, domains, and Alternative Model for Personality Disorders (AMPD) scores corresponded to the scores observed in the clinical population (Miller et al., 2022). In the main study, we observed a significant decrease in ISI scores after iCBT-I in the intervention group, −5.7 (*SE* = 0.8). Participants from the CAU group showed less improvement, −1.9 (0.7), *p* = 0.01, after 8 weeks (Pchelina et al., 2024).

**Table 1:** Predictors and outcome measures at baseline or pretreatment, overall and divided by group.

**Characteristic**	**All (*N* = 94)**	**iCBT-I + CAU (*N* = 53)**	**CAU (*N* = 41)**	**Statistic**
Age, years, median (IQR)	40 (32–52)	37 (28-−50)	41 (35-−59)	***U*** **=** **802.5;** ***p*** **=** 0.03^*^
Female, *n* (%)	54 (57.4)	30 (56.6)	24 (58.5)	χ^2^ =0.03; *p* = 0.85
Education, university, *n* (%)	79 (84.0)	42 (79.2)	37 (90.2)	χ^2^ = 5.49; *p* = 0.14
Second special	7 (7.4)	5 (9.4)	2 (4.9)	
Secondary	5 (5.3)	5 (9.4)	0 (0.0)	
Academic degree	3 (3.3)	1 (2)	2 (4.9)	
Social status, married	43 (45.7)	20 (37.7)	23 (56.1)	χ^2^ = 3.67; *p* = 0.30
Have a partner	20 (21.3)	12 (22.6)	8 (19.5)	
Divorced	8 (8.5)	6 (11.3)	2 (4.9)	
Single	23 (24.5)	15 (28.4)	8 (19.5)	
Employment status, employed, %	56 (59.5)	29 (54.7)	27 (65.8)	**χ^2^** **=** **10.01;** ***p*** **=** 0.02^*^
Unemployed	9 (10.0)	6 (11.3)	3 (7.4)	
Retired/disability	16 (17.0)	6 (11.3)	10 (24.4)	
Other	13 (13.5)	12 (22.7)	1 (2.4)	
Comorbidities, no	38 (40.4)	23 (43.4)	15 (36.6)	χ^2^ = 0.86; *p* = 0.84
Somatic or neurological	41 (43.5)	21 (39.6)	20 (48.8)	
Psychiatric	12 (12.8)	7 (13.2)	5 (12.2)	
Somatic/neurological and psychiatric	3 (3.3)	2 (3.8)	1 (2.4)	
Duration of insomnia, months, median (IQR)	36 (18–84)	36 (18–66.5)	54 (18.8–111)	*U* = 845.5; *p =* 0.38
Use of medications for insomnia, *n* (%)	79 (84.0)	47 (88.7)	32 (78.0)	χ^2^ = 1.95; *p =* 0.16
Use of benzodiazepines and/or Z-drugs, *n* (%)	31 (33.0)	19 (35.8)	12 (29.3)	χ^2^ = 0.45; *p =* 0.50
ISI, mean (*SD*)	14.4 (5.0)	15.4 (4.2)	13.1 (5.72)	***t*** **=** **2.11;** ***p** **=*** 0.04^*^
BDI-II, mean (*SD*)	10.5 (6.3)	10.7 (5.7)	10.3 (7.0)	*t* = 0.30; *p =* 0.76
BAI, mean (*SD*)	8.9 (7.2)	8.7 (6.9)	9.3 (7.6)	*t* =-0.37; *p =* 0.71
SF-12, mean (*SD*)	29.7 (6.5)	27.3 (5.6)	32.8 (6.2)	***t*** **=** **−4.4;** ***p*** < 0.0001^*^
DBAS, mean (*SD*)	100.4 (26.5)	101.2 (25.8)	99.3 (27.7)	*t* = 0.33; *p =* 0.74
LSC, mean (*SD*)	41.3 (12.6)	42.2 (11.8)	40.1 (13.6)	*t* = 0.80; *p =* 0.43
ESS, mean (*SD*)	4.4 (3.7)	4.8 (4.1)	4.0 (2.9)	*t* = 1.09; *p =* 0.28
SHI, mean (*SD*)	39.3 (12.1)	47.7 (8.2)	28.5 (6.3)	***t*** **=** **12.7;** ***p*** < 0.0001^*^
FSS, mean (*SD*)	37.2 (15.2)	36.2 (15.4)	38.3 (15.2)	*t* = −0.66; *p =* 0.51
SE, %, mean (*SD*)	77.5 (12.6)	75.4 (12.8)	80.4 (11.8)	*t* = −1.83; *p =* 0.07
SOL, min, mean (*SD*)	41.8 (31.9)	40.7 (28.7)	43.2 (36.2)	t = −0.34; *p =* 0.74
WASO, min, mean (*SD*)	36.1 (38.1)	40.5 (43.3)	30.0 (29.0)	t = 1.32; *p =* 0.19
TST, hours, mean (*SD*)	6.8 (1.4)	6.7 (1.6)	7.0 (1.1)	*t* = −1.22; *p =* 0.23
Number of completed modules	7 (1.7)	7.1 (1.6)	6.9 (1.9)	*t* = 0.38; *p =* 0.70
Number of sleep diaries	44.2 (11.8)	46.7 (10.7)	40.5 (12.5)	***t*** **=** **2.32;** ***p** **=*** 0.02^*^
Number of emails	9.1 (4.5)	9.4 (4.2)	8.7 (4.9)	*t* = 0.64; *p =* 0.52

ISI, Insomnia Severity Index; BDI-II, Beck Depression Inventory–II; BAI, Beck Anxiety Inventory; SF-12, quality-of-life 12-Item Short-Form Survey; FSS, Fatigue Severity Scale; ESS, Epworth Sleepiness Scale; DBAS, Dysfunctional Beliefs About Sleep Scale; LCS, Locus Control of Sleep Scale; SHI, Sleep Hygiene Index; SE, sleep effectiveness; SOL, sleep onset latency; WASO, wake after sleep onset; TST, total sleep time; iCBT-I, internet-based cognitive behavioral therapy for insomnia; CAU, care as usual; *SD*, standard deviation; IQR, interquartile range. Bold values denote statistical significance at the *p* < 0.05 level. ^*^denotes statistical significance at the *p* < 0.05 level.

### 3.1 Predictors of posttreatment ISI score improvement

**Figure 3:**
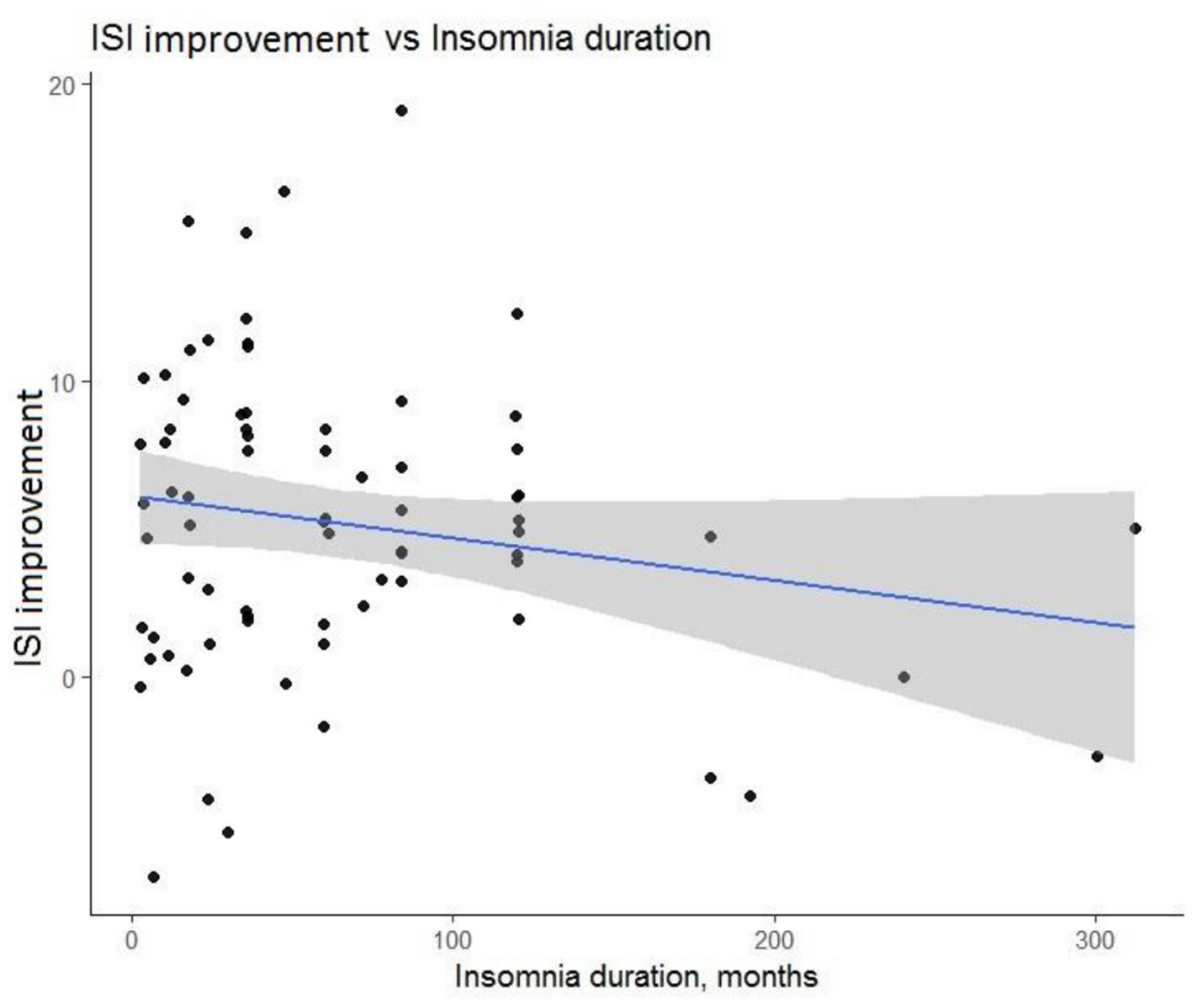
Prognostic value of duration of insomnia for Insomnia Severity Index (ISI) score improvement after treatment course.

**Figure 4:**
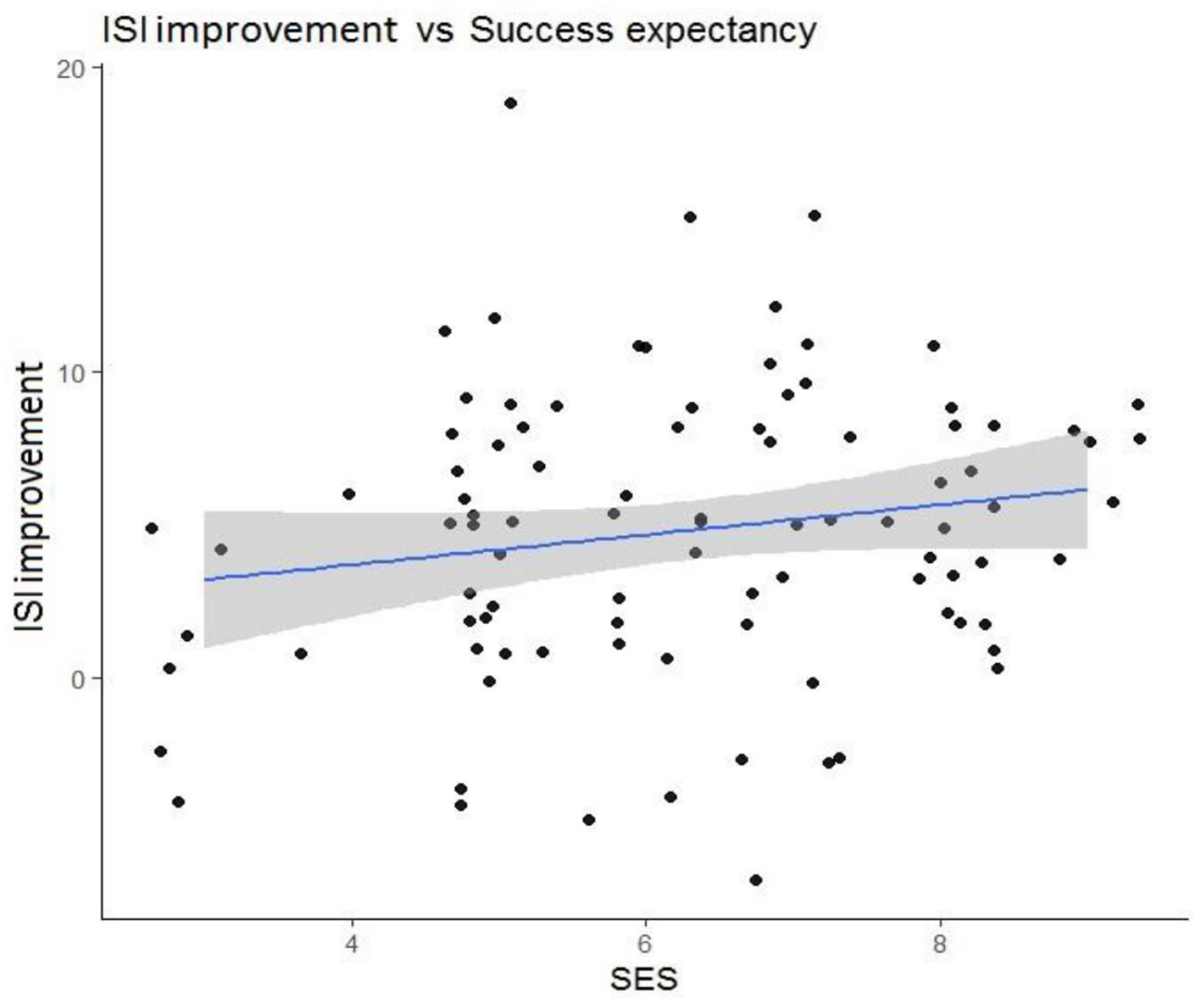
Prognostic value of success expectancy scale (SES) for Insomnia Severity Index (ISI) score improvement after treatment course.

Within the first set of multiple linear regression models, we examined predictors for posttreatment ISI score improvement (see Tables 2–4). Insomnia duration, *b* (*SE*) = −0.02 (0.01), *p* = 0.01, and success expectancy, *b* (*SE*) = 0.80 (0.27), *p* = 0.004, at baseline were significant predictors of posttreatment ISI improvement. For participants who had insomnia longer, improvement after iCBT-I was less prominent, although it has to be said that this effect was determined mainly by 17 observations of insomnia histories of 10 years and longer (see Figure 3). Participants who had a higher success expectancy were more likely to have a better iCBT-I effect posttreatment (see Figure 4). Models with success expectancy as a predictor were adjusted for insomnia duration, the number of completed modules, and the number of sleep diaries because these variables were considered to be potentially associated. The variance inflation factors in adjusted models were between 1.01 and 1.02, and no significant effects were found for these individual predictors, leading to the conclusion that the success expectancy is not explained by other independent variables in the model and its effect on the ISI score improvement does not depend on the level of another predictor. Significant predictors of insomnia improvement were the lower level of traits of attention seeking, *b* (*SE*) = −1.06 (0.51), *p* = 0.04; grandiosity, *b* (*SE*) = −1.50 (0.57), *p* = 0.01; distractibility, *b* (*SE*) = −1.57 (0.75), *p* = 0.04; and rigid perfectionism, *b* (*SE*) = −1.32 (0.65), *p* = 0.05 (Table 4).

**Table 2:** Single-predictor linear regression analysis with ISI score improvement or ISI response as dependent variables and demographic variables as potential predictors.

**Predictor**	**ISI score improvement**				**ISI response**			
	**Observed**		**Imputed**		**Observed**		**Imputed**	
	**Estimate (*SE*)**	***p***	**Estimate (*SE*)**	***p***	**Estimate (*SE*)**	***p***	**Estimate (*SE*)**	***p***
Intercept	−0.89 (1.48)	0.55	−1.71 (1.31)	0.20	−0.15 (0.15)	0.32	−0.71 (1.31)	0.20
ISI pretreatment	0.41 (0.10)	< 0.001^*^	0.45 (0.09)	< 0.001^*^	0.03 (0.01)	0.001^*^	0.45 (0.09)	< 0.001^*^
Group	−1.08 (1.07)	0.32	−0.66 (0.89)	0.46	−0.14 (0.11)	0.21	−0.15 (0.09)	0.10
Age	−0.01 (0.04)	0.71	0.001 (0.03)	0.97	0.001 (0.003)	0.72	0.001 (0.003)	0.61
Sex	−0.19 (1.02)	0.86	−0.47 (0.87)	0.59	−0.03 (0.11)	0.81	−0.07 (0.09)	0.40
Social status married (ref)	1.00 (0.00)		1.00 (0.00)		1.00 (0.00)		1.00 (0.00)	
Have a partner	−0.31 (1.32)	0.82	−0.18 (1.12)	0.12	0.01 (0.14)	0.97	−0.02 (0.12)	0.88
Divorced	−1.91 (1.98)	0.34	−2.58 (1.60)	0.59	−0.18 (0.20)	0.39	−0.14 (0.17)	0.39
Single	1.50 (1.24)	0.23	−1.57 (1.10)	0.07	−0.01 (0.13)	0.93	−0.02 (0.11)	0.85
Education university (ref)	1.00 (0.00)		1.00 (0.00)		1.00 (0.00)		1.00 (0.00)	
Second spec	1.34 (0.89)	0.48	0.84 (1.64)	0.61	0.36 (0.19)	0.06	0.33 (0.16)	0.05
Secondary	−2.61 (2.32)	0.27	−2.53 (1.93)	0.19	−0.22 (0.23)	0.35	−0.21 (0.20)	0.28
Academic degree	−3.09 (3.20)	0.34	−3.07 (2.45)	0.21	0.10 (0.32)	0.76	−0.07 (0.25)	0.78
Employment status Employed (ref)	1.00 (0.00)		1.00 (0.00)		1.00 (0.00)		1.00 (0.00)	
Unemployed	−0.78 (1.80)	0.67	0.37 (1.60)	0.82	−0.10 (0.19)	0.62	0.003 (0.16)	0.99
Retired/disability	−0.40 (1.42)	0.78	0.49 (1.20)	0.68	0.12 (0.15)	0.43	0.15 (0.12)	0.20
Other	0.43 (1.51)	0.78	−0.05 (1.30)	0.97	0.13 (0.15)	0.39	0.14 (0.13)	0.28
Comorbidities (no – ref)	1.00 (0.00)		1.00 (0.00)		1.00 (0.00)		1.00 (0.00)	
Somatic or neurological	−0.15 (1.11)	0.90	0.08 (0.95)	0.93	−0.002 (0.12)	0.98	−0.02 (0.10)	0.80
Psychiatric	−0.94 (1.56)	0.55	−0.72 (1.42)	0.61	−0.11 (0.16)	0.48	−0.08 (0.15)	0.58
Somatic/neurological and psychiatric	0.97 (4.64)	0.84	−1.65 (2.54)	0.52	0.41 (0.47)	0.38	−0.07 (0.26)	0.78
**Insomnia duration**	**−0.02 (0.01)**	**0.03** ^*^	**−0.02 (0.01)**	**0.01** ^*^	**−0.002 (0.001)**	**0.01** ^*^	**−0.002 (0.001)**	**0.01** ^*^
Concurrent pharmacotherapy no (ref)	1.00 (0.00)		1.00 (0.00)		1.00 (0.00)		1.00 (0.00)	
Yes	1.34 (1.41)	0.37	0.94 (1.18)	0.42	0.01 (0.15)	0.94	0.08 (0.12)	0.53
Concurrent therapy with benzodiazepines, no, (ref)	1.00 (0.00)		1.00 (0.00)		1.00 (0.00)		1.00 (0.00)	
Yes	−0.22 (1.06)	0.84	0.19 (0.92)	0.84	−0.06 (0.11)	0.61	0.01 (0.09)	0.95
Concurrent therapy with antidepressants, no, (ref)	1.00 (0.00)		1.00 (0.00)		1.00 (0.00)		1.00 (0.00)	
Yes	0.49 (1.02)	0.64	−0.11 (0.89)	0.90	0.001 (0.11)	0.99	−0.02 (0.09)	0.83
Concurrent psychotherapy, no (ref)	1.00 (0.00)		1.00 (0.00)		1.00 (0.00)		1.00 (0.00)	
Yes	0.54 (1.20)	0.65	−0.21 (1.02)	0.84	−0.12 (0.12)	0.34	−0.06 (0.10)	0.59
**Success expectancy**	**0.87 (0.30)**	**0.005** ^*^	**0.80 (0.27)**	**0.004** ^*^	**0.09 (0.03)**	**0.003** ^*^	**0.08 (0.03)**	**0.003** ^*^

Estimates, standard errors, and *p*-values are presented for the main effect of the analyzed variable in a separate model. The greater ISI score improvement reflecting improvement after treatment is characterized by the higher estimate. ISI, Insomnia Severity Index; estimate (*SE*), estimated mean difference (standard error). Bold values denote statistical significance at the *p* < 0.05 level. ^*^denotes statistical significance at the *p* < 0.05 level.

**Table 3:** Single-predictor linear regression analysis with ISI score improvement or ISI response as dependent variables and pretreatment questionnaires as potential predictors.

**Predictor**	**ISI score improvement**				**ISI response**			
	**Observed**		**Imputed**		**Observed**		**Imputed**	
	**Estimate (*SE*)**	***p***	**Estimate (*SE*)**	***p***	**Estimate (*SE*)**	***p***	**Estimate (*SE*)**	***p***
Intercept	−0.89 (1.48)	0.55	−1.71 (1.31)	0.20	−0.15 (0.15)	0.32	−0.71 (1.31)	0.20
ISI	0.41 (0.10)	< 0.001^*^	0.45 (0.09)	< 0.001^*^	0.03 (0.01)	0.001^*^	0.45 (0.09)	< 0.001^*^
Beck Anxiety Inventory	−0.09 (0.07)	0.24	−0.12 (0.06)	0.07	−0.01 (0.01)	0.41	−0.01 (0.01)	0.11
Beck Depression Inventory–II	−0.04 (0.10)	0.69	−0.09 (0.08)	0.27	−0.005 (0.01)	0.65	−0.01 (0.01)	0.28
Quality-of-life 12-Item Short-Form Survey	0.05 (0.08)	0.55	0.02 (0.07)	0.75	−0.01 (0.01)	0.42	−0.01 (0.01)	0.41
Fatigue Severity Scale	0.002 (0.04)	0.96	−0.01 (0.03)	0.83	0.002 (0.003)	0.59	0.001 (0.003)	0.81
Epworth Sleepiness Scale	0.07 (0.14)	0.63	0.13 (0.12)	0.27	−0.001 (0.01)	0.96	0.005 (0.01)	0.70
Dysfunctional Beliefs About Sleep Scale	−0.01 (0.02)	0.68	0.004 (0.02)	0.79	−0.001 (0.002)	0.68	0.0002 (0.002)	0.88
Locus Control of Sleep Scale	−0.004 (0.04)	0.93	0.04 (0.03)	0.22	−0.001 (0.004)	0.83	0.001 (0.004)	0.76
**Sleep hygiene index**	0.04 (0.04)	0.29	0.03 (0.03)	0.38	***0.01 (0.04)***	***0.05***	**0.01 (0.003)**	**0.02** ^*^
**Sleep effectiveness**	−0.03 (0.04)	0.49	−0.004 (0.04)	0.92	**0.80 (0.27)**	**0.004** ^*^	−0.002 (0.004)	0.54
Sleep quality	1.90 (1.34)	0.16	0.98 (0.33)	0.33	0.09 (0.14)	0.49	0.03 (0.10)	0.73
Sleep onset latency	−0.004 (0.02)	0.79	< -0.001 (0.01)	0.98	−0.002 (0.002)	0.27	−0.001 (0.001)	0.52
Wake after sleep onset	0.01 (0.01)	0.58	−0.01 (0.01)	0.58	0.001 (0.001)	0.68	0.002 (0.001)	0.17
Total sleep time	−0.11 (0.39)	0.78	0.31 (0.33)	0.34	0.03 (0.04)	0.41	−0.004 (0.03)	0.89
Number of completed sleep diaries	0.02 (0.05)	0.67	0.06 (0.04)	0.14	0.001 (0.01)	0.92	0.003 (0.004)	0.44
Number of completed modules	0.16 (0.31)	0.60	0.31 (0.23)	0.18	−0.02 (0.03)	0.49	0.004 (0.02)	0.85
Number of emails from patient	−0.06 (0.13)	0.66	0.04 (0.10)	0.71	0.01 (0.01)	0.55	0.01 (0.01)	0.17

Estimates, standard errors, and *p*-values are presented for the main effect of the analyzed variable in a separate model. The greater ISI score improvement reflecting improvement after treatment is characterized by the higher estimate. ISI, Insomnia Severity Index; Estimate (*SE*), estimated mean difference (standard error). Bold values denote statistical significance at the *p* < 0.05 level. Italic values denote nearly statistical significance at the *p* = 0.05. ^*^denotes statistical significance at the *p* < 0.05 level.

**Table 4:** Single-predictor linear regression analysis with ISI improvement or ISI response as dependent variables and personality traits as potential predictors.

**Predictor**	**ISI improvement**				**ISI Response**			
	**Observed**		**Imputed**		**Observed**		**Imputed**	
	**Estimate (*SE*)**	***p***	**Estimate (*SE*)**	***p***	**Estimate (*SE*)**	***p***	**Estimate (*SE*)**	***p***
Intercept	−0.89 (1.48)	0.55	−1.71 (1.31)	0.20	−0.15 (0.15)	0.32	−0.71 (1.31)	0.20
ISI pretreatment	0.41 (0.10)	< 0.001^*^	0.45 (0.09)	< 0.001^*^	0.03 (0.01)	0.001^*^	0.45 (0.09)	< 0.001^*^
Anxiousness	−0.36 (0.61)	0.56	−0.59 (0.55)	0.29	−0.01 (0.06)	0.82	−0.02 (0.06)	0.67
Attention seeking	−0.76 (0.58)	0.20	−1.06 (0.51)	0.04^*^	−0.11 (0.06)	0.06	−0.11 (0.05)	0.05^*^
Callousness	0.002 (1.30)	0.999	0.47 (1.10)	0.67	−0.10 (0.13)	0.47	−0.11 (0.11)	0.34
Deceitfulness	−0.69 (1.08)	0.52	−0.29 (0.96)	0.76	−0.08 (0.11)	0.46	−0.10 (0.10)	0.34
Depressivity	−0.10 (1.34)	0.94	0.67 (1.12)	0.55	−0.10 (0.14)	0.50	−0.09 (0.11)	0.43
Distractibility	−1.37 (0.65)	0.04^*^	−1.50 (0.57)	0.01^*^	−0.17 (0.06)	0.01^*^	−0.18 (0.06)	0.004^*^
Eccentricity	−0.33 (0.67)	0.63	−0.43 (0.59)	0.47	−0.07 (0.07)	0.32	−0.08 (0.06)	0.19
Emotional lability	−1.35 (0.79)	0.09	−1.16 (0.73)	0.12	−0.16 (0.08)	0.05.	−0.17 (0.08)	0.02^*^
Grandiosity	−0.76 (0.92)	0.41	−1.57 (0.75)	0.04^*^	−0.01 (0.09)	0.92	−0.10 (0.08)	0.20
Hostility	−0.50 (0.80)	0.53	−0.29 (0.71)	0.69	−0.11 (0.09)	0.19	−0.12 (0.07)	0.11
Impulsivity	0.41 (0.82)	0.62	0.14 (0.73)	0.84	−0.03 (0.08)	0.74	−0.05 (0.08)	0.51
Intimacy avoidance	−0.23 (0.68)	0.74	0.21 (0.61)	0.73	−0.02 (0.07)	0.80	−0.01 (0.06)	0.87
Irresponsibility	−1.18 (1.30)	0.37	−1.86 (1.15)	0.11	−0.16 (0.13)	0.24	−0.19 (0.12)	0.12
Manipulativeness	−0.76 (0.97)	0.44	−1.04 (0.84)	0.22	−0.14 (0.10)	0.15	−0.16 (0.09)	0.07
Perceptual dysregulation	−0.61 (1.59)	0.70	−0.82 (1.40)	0.56	−0.05 (0.17)	0.74	−0.09 (0.15)	0.56
Perseveration	0.14 (0.81)	0.86	−0.50 (0.68)	0.46	−0.03 (0.08)	0.74	−0.08 (0.07)	0.28
Restricted affectivity	−1.83 (0.87)	0.04^*^	−0.83 (0.77)	0.29	−0.23 (0.09)	0.01^*^	−0.17 (0.08)	0.04^*^
Rigid perfectionism	−1.21 (0.73)	0.10	−1.32 (0.65)	0.05	−0.11 (0.08)	0.16	−0.13 (0.07)	0.05
Risk taking	−1.13 (0.99)	0.26	−1.31 (0.73)	0.08	−0.15 (0.10)	0.13	−0.18 (0.07)	0.02^*^
Separation insecurity	−1.00 (0.68)	0.15	−0.98 (0.62)	0.12	−0.11 (0.07)	0.13	−0.12 (0.06)	0.06
Submissiveness	0.62 (0.72)	0.39	0.01 (0.65)	0.99	−0.06 (0.08)	0.45	−0.04 (0.07)	0.52
Suspiciousness	−0.84 (0.94)	0.38	−0.86 (0.80)	0.28	−0.08 (0.10)	0.44	−0.06 (0.08)	0.46
Unusual beliefs experience	−0.78 (1.57)	0.62	−0.89 (1.45)	0.54	−0.15 (0.16)	0.36	−0.15 (0.15)	0.31
Withdrawal	−0.57 (0.74)	0.44	−0.23 (0.65)	0.72	−0.15 (0.07)	0.04^*^	−0.13 (0.07)	0.05^*^

Estimates, standard errors and *p*-values are presented for the main effect of the analyzed variable in a separate model. The greater ISI improvement reflecting improvement after treatment is characterized by the higher estimate. ISI, Insomnia Severity Index; Estimate (*SE*), estimated mean difference (standard error). ^*^denotes statistical significance at the *p* < 0.05 level.

### 3.2 Predictors of response

The results of the multiple linear regression models for ISI score improvement were further confirmed with logistic regression for response as the dependent variable. The following baseline variables had a significant predictive effect on the response: insomnia duration, *b* (*SE*) = −0.002 (0.001), *p* = 0.01, and success expectancy, *b* (*SE*) = 0.08 (0.03), *p* = 0.003. Another predictor of response was the higher sleep hygiene index pretreatment, *b* (*SE*) = 0.01 (0.003), *p* = 0.02. The following personality traits significantly predicted response: distractibility, *b* (*SE*) = −0.18 (0.06), *p* = 0.004; emotional liability, *b* (*SE*) = −0.17 (0.08), *p* = 0.02; restricted affectivity, *b* (*SE*) = −0.17 (0.08), *p* = 0.04; risk-taking, *b* (*SE*) = −0.18 (0.07), *p* = 0.02; and withdrawal, *b* (*SE*) = −0.13 (0.07), *p* = 0.05. Neither the number of completed modules, the number of completed sleep diaries, nor the number of emails sent to the iCBT-I specialist during the program had a significant effect on the outcomes. Tables 2–4 display the results of the logistic regression, with response as the outcome.

### 3.3 Sensitivity analysis

In completers' data analysis, we observed similar prediction effects on ISI score improvement as in imputed data analysis across all demographic and medical history predictors and most of the self-report questionnaires. Only the attention-seeking and grandiosity personality traits were not significant predictors of ISI score improvement before imputation, with *p*-values of 0.20 and 0.41, respectively; however, after imputation, they became significant (*p* = 0.04 and.04, respectively). Restricted affectivity was a significant predictor of ISI score improvement in the complete case analysis (*p* = 0.04) but became non-significant after (*p* = 0.29). The SHI score, attention seeking, emotional lability, and risk-taking were not significant predictors of response before imputation, with *p*-values of 0.05, 0.06, 0.05, and 0.13, respectively, but became significant predictors after imputation (*p*s = 0.02, 0.05, 0.02, and 0.02, respectively). In contrast, SE, a significant predictor of response before the imputation (*p* = 0.004), became non-significant after imputation, with a *p*-value of 0.54. SE was one of the imputed variables that had 10% of its data missing. Missing data are explained by participants' unwillingness to fill in the iCBT-I sleep diary, which was used to obtain weekly average subjective sleep characteristics during the last week of the iCBT-I course.

## 4 Discussion

In the present study, we aimed to identify characteristics of patients with CI who improved after iCBT-I. We found that a longer duration of insomnia before the start of the therapy was associated with worse outcomes. This is in line with other studies showing that a longer insomnia duration is associated with poorer outcomes in face-to-face and online CBT-I interventions (Pchelina et al., 2023; Van Houdenhove et al., 2011). One explanation could be that a long duration of sleep problems is associated with lower success expectancy after years of unsuccessful attempts to fix the problem; however, further analysis of the adjusted model did not show a significant interaction effect between insomnia duration and success expectancy. The subgroup of patients with long-term insomnia may be eligible for individually tailored CBT-I administered by qualified psychotherapists, who can address issues of mistrust and low motivation to follow recommendations. Another significant predictor of the effect was success expectancy. This finding is consistent with many studies on the association between treatment expectancy and mental health outcomes, although the specific link is not explicitly addressed in the studies of iCBT effectiveness for insomnia (Constantino et al., 2018). However, another study of predictors of the effect of cognitive behavioral therapy for chronic insomnia in breast cancer survivors has shown that higher initial levels of treatment expectancies significantly predicted subjective sleep improvement (Tremblay et al., 2009). Many authors link success expectancy with better adherence, known to be an important mediator of the effect of iCBT-I (Matthews et al., 2013; Horsch et al., 2015). However, this association was not observed in our study, and neither of the adherence measures we used predicted the treatment outcome. It should be noted that the measures of adherence, investigated in this study, reflected only aspects of technological utilization of the program, while the sleep diary–derived measures of adherence (e.g., compliance with the bedtime and waketime routine, extent of bedtime restriction according to the bedtime restriction technique, and consistency in implementing the recommendations) could be more significant mediators.

Several facet traits were predictors of outcomes. The distractibility personality facet or difficulty in maintaining focus and easily getting distracted by external stimuli can be a sign of hyperarousal, which interferes with sleep initiation and maintenance as well as daily activities reflected in PID-5. In line with this, one study has shown that insomnia complaints were associated with a reduced capacity to control attention to negative stimuli (Nota and Coles, 2018). To our knowledge, personality facets such as attention seeking and grandiosity were never found to be associated with the outcome in any way. It should be noted that all effects of personality traits were negative in contrast to one previous study, in which a higher level of perfectionism facilitated the effect of iCBT-I (Johann et al., 2023). Authors have theorized that perfectionism is an adaptive trait that helps people strictly comply with a set of rules of CBT-I to strengthen their conditioned association between the bed and sleep. Conversely, maladaptive perfectionism may cause hyper-organization, with the high standards of regimen and environment for sleep, leading people to worry about possible mistakes, further affecting their sleep.

The present study has some important limitations to consider. Our study is *post-hoc*, meaning that the sample size was calculated for the primary iCBT-I effectiveness study to detect small to medium effect sizes. This makes our secondary analysis underpowered because predictor effects in internet-based interventions tend to be small. As in other *post-hoc* analyses, our study is subject to the problem of multiple comparisons, which can increase the chance of incidental findings. Therefore, our results must be considered exploratory and verified in an appropriately designed study. On one hand, the fact that the effects observed for the continuous outcome are largely replicated in the categorical outcome demonstrates the robustness of the analysis. One strength of the present study is that all participants had unrestricted access to the health care resources, making the results more generalizable. On the other hand, it might have resulted in a smaller effect of iCBT-I on insomnia severity (Pchelina et al., 2024). Consequently, our predictor analyses could have been additionally underpowered. Moreover, the participants in the delayed treatment group had significantly lower pretreatment ISI scores, which were due to the positive dynamic in the CAU group and could be explained by access to all kinds of treatment. Although the mean score of the ISI was still above normal, this subsample could have been already less burdened at pretreatment and less representative of people with insomnia. For this reason, we adjusted all the models for the pretreatment scores on the ISI. Because of the small sample size, we did not perform a moderator analysis to identify for whom and under what conditions treatments have different effects.

## 5 Conclusion

The low and indistinct effects found in this analysis do not provide definitive answers regarding the predictors of insomnia. Instead, they serve to augment the existing literature. The study demonstrates possible associations between patient characteristics and iCBT-I treatment outcomes. This evidence gives insights into the decision-making process when a clinician determines whether to refer a patient to iCBT-I or a CBT expert. In particular, it shows that attitudes toward treatment and the duration of insomnia history probably impact a person's motivation to follow CBT-I recommendations and, consequently, their effect. This finding justifies the need for carefully collecting patients' medical histories and strengthening their expectations of the therapy. Several personality characteristics were predictive of treatment outcomes as well. A personalized approach to treatment selection will enhance the quality of care and help patients achieve remission faster. However, further studies are needed to confirm the associations between patients' characteristics and treatment outcomes of iCBT-I.

## Data Availability

The raw data supporting the conclusions of this article will be made available by the authors, without undue reservation.
